# Automated Indirect Immunofluorescence Evaluation of Antinuclear Autoantibodies on HEp-2 Cells

**DOI:** 10.1155/2012/651058

**Published:** 2012-11-11

**Authors:** Jörn Voigt, Christopher Krause, Edda Rohwäder, Sandra Saschenbrecker, Melanie Hahn, Maick Danckwardt, Christian Feirer, Konstantin Ens, Kai Fechner, Erhardt Barth, Thomas Martinetz, Winfried Stöcker

**Affiliations:** ^1^Institute for Experimental Immunology, Euroimmun AG, Seekamp 31, 23560 Lübeck, Germany; ^2^Institute for Neuro- and Bioinformatics, University of Lübeck, Ratzeburger Allee 160, 23562 Lübeck, Germany

## Abstract

Indirect immunofluorescence (IIF) on human epithelial (HEp-2) cells is considered as the gold standard screening method for the detection of antinuclear autoantibodies (ANA). However, in terms of automation and standardization, it has not been able to keep pace with most other analytical techniques used in diagnostic laboratories. Although there are already some automation solutions for IIF incubation in the market, the automation of result evaluation is still in its infancy. Therefore, the EUROPattern Suite has been developed as a comprehensive automated processing and interpretation system for standardized and efficient ANA detection by HEp-2 cell-based IIF. In this study, the automated pattern recognition was compared to conventional visual interpretation in a total of 351 sera. In the discrimination of positive from negative samples, concordant results between visual and automated evaluation were obtained for 349 sera (99.4%, kappa = 0.984). The system missed out none of the 272 antibody-positive samples and identified 77 out of 79 visually negative samples (analytical sensitivity/specificity: 100%/97.5%). Moreover, 94.0% of all main antibody patterns were recognized correctly by the software. Owing to its performance characteristics, EUROPattern enables fast, objective, and economic IIF ANA analysis and has the potential to reduce intra- and interlaboratory variability.

## 1. Introduction

The detection of autoantibodies against the cell nuclei (ANA) and cytoplasmic components plays an important role in the diagnosis of many autoimmune diseases, such as systemic lupus erythematosus, mixed connective tissue disease, rheumatoid arthritis, progressive systemic sclerosis, dermato-/polymyositis, Sjögren's syndrome, and chronic active autoimmune hepatitis. The prevalence of ANA varies between 20 and 100%, depending on the disease and type of antibody [1–4].

The gold standard for ANA screening is indirect immunofluorescence (IIF) on human epithelial (HEp-2) cells [5–7]. Displaying a multitude of authentic autoantigens, this antigenic substrate enables highly sensitive preidentification of autoantibodies by their characteristic fluorescence patterns [8], and the determination of their titers. In addition, the confirmation of positive screening results and the identification of single ANA specificities by monospecific immunoassays (e.g., enzyme-linked immunosorbent assay (ELISA) or immunoblot) are recommended to support differential diagnosis, disease monitoring, and prognostic assessment. This two-step strategy has been challenged by automated ELISA and multiplex approaches promising easy, cost-effective high-throughput performance and standardization [9, 10]. However, these assays may produce inaccurate (false negative) screening results, mainly because the number of displayed purified or recombinant antigens is limited, or, when using nuclear homogenates as substrate, relevant epitopes may be altered or lost during the process of solid-phase coating [5, 6, 11–15]. 

As mentioned before, HEp-2-cell-based IIF is the method of choice for ANA screening. Although there are some automation solutions for IIF incubation about to be launched on the market, the evaluation is still carried out visually by laboratory technicians, thus being time consuming, subjective, error prone, and contributive to inter-observer variability. This, together with the growing demand for ANA testing, reinforces the need for automation and standardization of IIF evaluation. So far, only a few more or less advanced commercial platforms based on automated motorized camera-microscopes and digital image analysis software have been introduced [16–22].

In the current study, we evaluated a novel system (EUROPattern Suite) for largely automated processing of IIF slides, and the recording and interpretation of immunofluorescence images of HEp-2 cells. The performance of this novel system was compared to visual IIF interpretation, focusing on positive/negative classification and pattern recognition. 

## 2. Materials and Methods

### 2.1. Human Sera

Two sample collectives were examined. Collective A consisted of 200 consecutive serum samples submitted to a reference laboratory (Lübeck, Germany) for routine ANA testing. Empirically, the majority of these samples tend to show complicated mixed patterns, whereas only a few of them are negative. Collective B comprised 151 serum samples originating from different referral laboratories, including 44 samples from patients with systemic rheumatic disease (10 systemic lupus erythematosus, 10 systemic sclerosis, 16 Sjögren's syndrome, 8 dermato-/polymyositis), 12 samples with specific ANA or anticytoplasmatic autoantibodies, 47 samples from disease controls, and 48 samples from healthy blood donors. The samples were blinded for analysis. All study procedures were approved by the local ethics committee. 

### 2.2. Indirect Immunofluorescence (IIF) Assay

ANA detection was performed by IIF using HEp-2 cells (Euroimmun, Lübeck, Germany). The cells were coated onto cover slips, fixed with acetone, cut into fragments (biochips), and glued onto microscope slides. The complete incubation process was carried out manually: serum samples diluted 1 : 100 were incubated with the HEp-2 cell substrate for 30 minutes at room temperature. After washing with PBS-Tween, the slides were incubated for another 30 minutes with goat anti-human IgG conjugated with fluorescein isothiocyanate plus propidium iodide for counterstaining (Euroimmun) to label specifically bound antibodies. After a second washing step and embedding, the slides were evaluated. 

### 2.3. Evaluation of Antinuclear Autoantibodies

IIF slides were subjected to automated immunofluorescence microscopy (as described below), with the system taking focused images of all reaction fields. Subsequently, using the same images, the fluorescence patterns were evaluated in two ways: (i) by the EUROPattern software (Euroimmun) and (ii) visually by two laboratory technicians who worked independently without reference to the other's and the software's readings. Sera with an antibody titer equal to or greater than 1 : 100 were considered as positive. Based on HEp-2 cells, the following patterns were reported: homogenous, speckled, nucleolar, nuclear dots, centromeres, cytoplasmic, and negative.

### 2.4. Automated Processing

The EUROPattern Suite (Euroimmun) consists of an automated EUROPattern Microscope, the laboratory management software EUROLabOffice, and the pattern recognition software EUROPattern.

**Figure 1:**
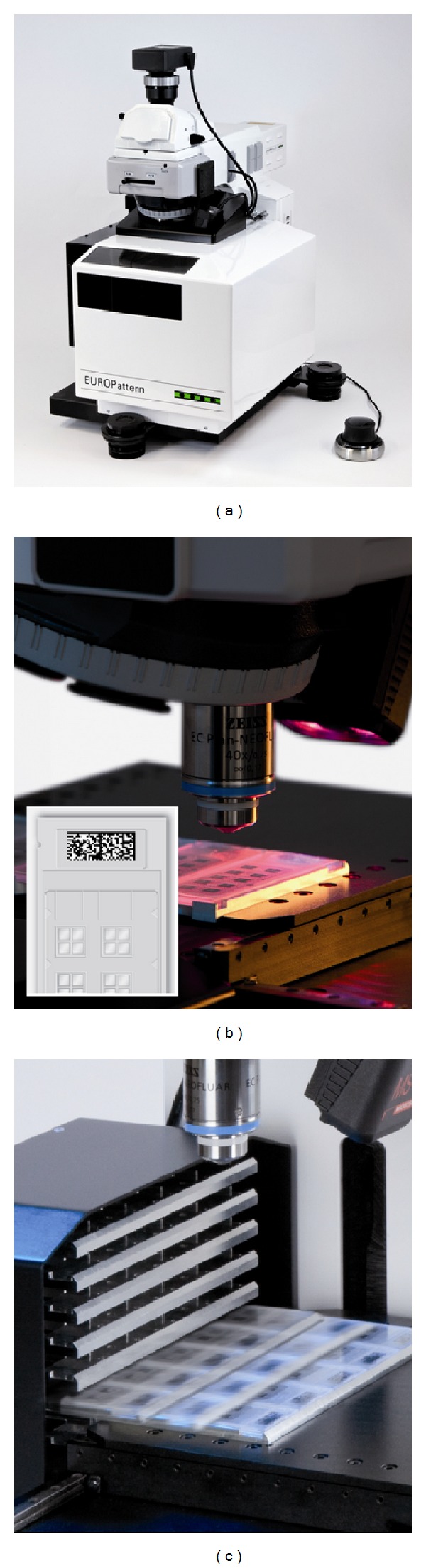
EUROPattern Microscope for automated acquisition of high-resolution immunofluorescence images. (a) Motorized microscope including camera, controlled LED, microscope control device, optional eyepieces, (b) matrix code reader, and (c) slide magazine. The microscope stage and the magazine are surrounded by a casing keeping out the sunlight and protecting the substrate fluorescence from fading. The microscope is part of the EUROPattern Suite, which additionally contains a laboratory management software (EUROLabOffice) and an automatic pattern recognition software (EUROPattern).

The specialized EUROPattern Microscope has been tailored to the requirements of automated IIF. As a motorized camera-microscope, it provides automated acquisition of high-resolution immunofluorescence images. It contains a slide magazine with a capacity for 500 reaction fields and a matrix code scanner enabling slide identification. Instead of conventional illumination fittings it has a controlled LED, which maintains a constant light flux (>50,000 hrs), ensuring highly reproducible results (Figure 1).

EUROLabOffice supports IIF processing by data exchange with a central Laboratory Information System (LIS), automatic protocol generation, interconnection with further laboratory devices (e.g., dilution/incubation systems), and data connection with EUROPattern for archiving of IIF images and automatic image interpretation. The software consolidates all the results from IIF and other analytical techniques (ELISA, immunoblot, and radioimmunoprecipitation assay) into one report per patient and provides a validation process.

EUROPattern is a fast and comprehensive IIF pattern recognition system providing objective test results. As a closed system, EUROPattern requires specific HEp-2 or HEp-20-10 cell-based test kits (Euroimmun) with a particular anti-human IgG conjugate enabling image segmentation by counterstaining. The software identifies the cells, calibrates the image, classifies the image as negative or positive, and, in case of a positive result, extracts 179 features and identifies the pattern(s). The classification is based on k-nearest neighbour algorithm with a reference database of more than 5,000 images (115,000 cell references) and rule-based synthesizing of cell results to one result per dilution. Single as well as mixed patterns can be identified. If a sample has been incubated in different dilutions, EUROPattern additionally merges the results of the different images into one patient report containing the patterns and estimated antibody titer.

**Figure 2:**
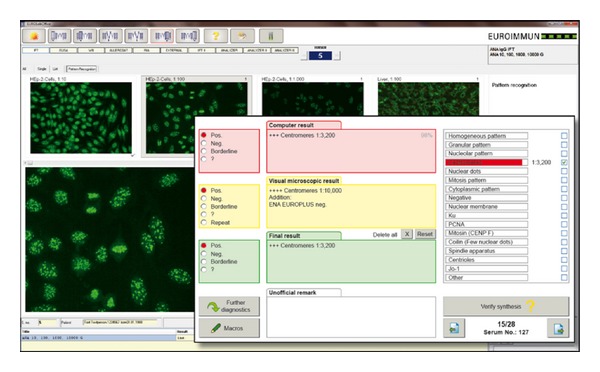
Graphical user interface of EUROPattern. For each patient sample, EUROPattern displays the images of different sample dilutions, preliminary or additional results, and the software-generated result (recognized pattern, antibody titer, and calculated confidence value) on one report form. The software-generated result can be confirmed by mouse click or, if necessary, modified and specified using a readily displayed list of fluorescence patterns.

EUROPattern is a computer-aided diagnostic system, meaning that all automatically retrieved results have to be validated by the laboratory staff in the Graphical User Interface (GUI), which is plugged into EUROLabOffice. For an efficient laboratory process, all images with a negative result can be displayed in a list, ordered by a normalized image fluorescence intensity. If deemed necessary, the positive/negative cutoff may be corrected by mouse click and all remaining negatives can be validated as well in one step. All positive results can be reviewed patient by patient. For each patient, the EUROPattern GUI displays the images of different sample dilutions and the consolidated results, including the identified patterns with the corresponding estimated titer and the calculated confidence value. The automatically generated result can be further detailed using a readily displayed list of antibody patterns (Figure 2).

### 2.5. Statistics

The degree of interrater agreement between visual and automated antibody pattern interpretation was assessed by the percentage of concordance and by kappa coefficients. According to Altman [23], kappa (*κ*) values were interpreted as follows: ≤0.20 poor, 0.21–0.40 fair, 0.41–0.60 moderate, 0.61–0.80 good, and 0.81–1.00 very good agreement. Statistical analyses were carried out using GraphPad QuickCalcs (GraphPad Software Inc., La Jolla, CA, USA).

## 3. Results

### 3.1. Positive/Negative Classification

The efficient usage of any automated ANA detection system requires first of all a reliable identification of negatives. Therefore, the capability of the EUROPattern system to differentiate negative from positive samples was analyzed.

About 40 installations of EUROPattern in different laboratories worldwide have revealed that visual IIF analysis remains partially subjective, resulting in the requirement to keep the fluorescence intensity cutoff configurable. This classificator setting is part of an optimization process during the introduction in immunologic laboratories to set the relation of sensitivity to specificity. As an approach to standardization, a recommended basic setup is available for EUROPattern that has been chosen for the evaluation of the EUROPattern classificator.

Out of a total of 200 sera sent to a reference laboratory for routine ANA testing (collective A), 193 sera were classified as antibody positive both by visual and automated evaluation. Out of 7 sera tested negative by visual examination, 6 were negative in EUROPattern, whereas one sample was reported positive with cytoplasmic fluorescence. 

Out of 151 sera from rheumatic patients and controls (collective B), 79 sera were assessed as positive and 71 as negative both by visual and automated examination. There was one discrepant serum that was negative by visual evaluation, but demonstrated faint positive fluorescence (low probability rate) according to EUROPattern.

Referring to the total of 351 samples, there was an agreement of 99.4% (*κ* = 0.984) between the visual and automated approach regarding positive/negative discrimination. The analytical sensitivity and specificity of EUROPattern amounted to 100% and 97.5%, respectively, while the positive and negative predictive value were 99.3% and 100%, respectively (Table 1).

### 3.2. Pattern Recognition

**Figure 3:**
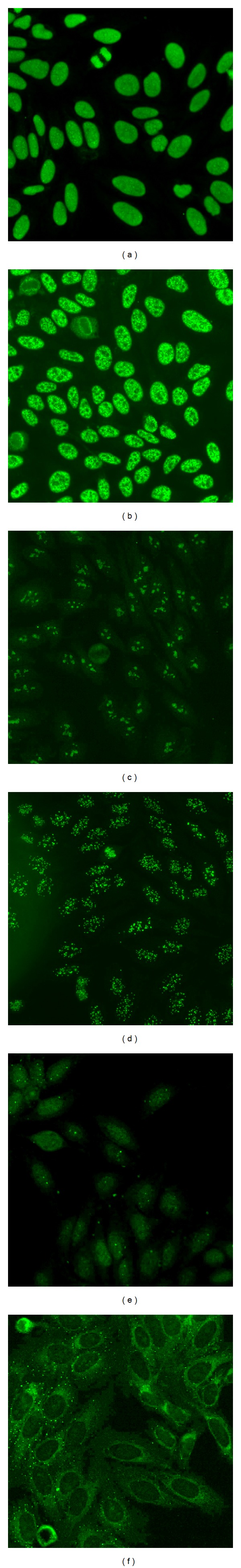
Representative indirect immunofluorescence patterns of antinuclear and anticytoplasmic autoantibodies on HEp-2 cells: (a) homogenous, (b) speckled, (c) nucleolar, (d) centromeres, (e) nuclear dots, and (f) cytoplasmic.

In daily routine with EUROPattern, after the one-step validation of the samples classified as negative, all remaining positive samples have to be validated by the laboratory staff patient by patient. We analyzed the ability of EUROPattern for recognition of homogenous, speckled, nucleolar, centromere, nuclear dotted, cytoplasmic, and negative patterns (Figure 3).

In collective A, correct and complete pattern recognition (including mixed patterns) was observed in 49.0% of the samples (47.7% of positive samples). In 93.0% of the samples (93.3% of positive samples) at least the main pattern was recognized correctly.

In collective B, correct and complete pattern recognition (including mixed patterns) was observed in 74.2% of the samples (51.9% of positive samples). In 96.7% of the samples (94.9% of positive samples) at least the main pattern was recognized correctly.

Referring to the total of 351 serum samples, the automatically retrieved results were correct and complete (including mixed patterns) in 59.8% of all samples (48.9% of positive samples). The overall efficiency of automated main pattern recognition was 94.0% and varied for the different patterns, declining in the following order: centromeres, nuclear dots (100%) > negative (97.5%) > nucleolar (95.6%) > speckled (94.6%) > cytoplasmic (93.1%) > homogenous (81.8%). In 21 out of 351 (6.0%) sera, the main pattern was not recognized (Table 2).

## 4. Discussion

The automation of IIF processing and evaluation is a major step towards standardization of the method. Standardization is a crucial concern because of intra- and interlaboratory variations, which may have methodological causes (e.g., microscope, type and running time of the microscope bulb, test kits, reagents, and incubation method/device) but can also be influenced by subjective image interpretation, individual expertise, and experience of the laboratory staff. Systems for automated IIF evaluation may contribute to the reduction of errors and pave the way to standardized ANA testing [16, 21].

In this study, we compared the results of classical visual reading with automated pattern recognition by EUROPattern for 351 samples. None of the 272 positive patient samples were missed out, and 77 out of 79 negatives were identified as negative by the software. Based on a 99.4% agreement with visual interpretation, EUROPattern proved highly capable of performing reliable positive/negative discrimination of IIF results. In comparison, for the AKLIDES software (Medipan, Berlin, Germany) an agreement with visual positive/negative discrimination of 90.0 to 98.9% was reported [17, 18, 20, 21]. The agreement rate of the NOVA View system (Inova, San Diego, USA) amounted to 92.2% [22]. 

In EUROPattern, the automatically determined patterns were correct and complete in 210 out of 351 cases and correct and meaningful but not complete (“main pattern”) in another 120 cases, enabling main pattern recognition in 94.0% of cases. The lowest performance in pattern recognition was found for the homogenous pattern type (81.8%), while the performance rates for the other patterns ranged between 93.1 and 100%. This finding may be due to the fact that many investigators tend to interpret a dense granular pattern as a homogenous pattern, while the automated system reports a granular pattern. Moreover, the software-based recognition of a homogenous staining may be impaired by a superimposed granular nuclear or cytoplasmic fluorescence in samples with a mixed pattern.

Inadvertently, sera with an anticentromere or antinuclear dot pattern were underrepresented in the present study. However, in preliminary studies, we evaluated the EUROPattern system by use of 23 samples demonstrating an anti-centromere pattern and 32 samples demonstrating an anti-nuclear dot pattern by visual HEp-2 cell interpretation. EUROPattern identified the respective pattern in 19/23 and 26/32 sera, corresponding to recognition rates of 82.6% (anti-centromere) and 81.3% (anti-nuclear dot). Discrepant assessment was found for 10 samples in which additional staining patterns, such as strong cytoplasmic fluorescence, interfered with the automated assessment.

The recognition of mixed patterns is a critical point in IIF ANA detection, because dominant autoantibodies (or unspecific antibodies) may mask another diagnostically relevant autoantibody or complicate pattern differentiation. As also reported for the AKLIDES system [17, 21], distinction of patterns with two or more autoantibodies can be difficult, depending on their number, target, and titer. This point is also reflected by the present study, showing correct and complete pattern recognition (including mixed patterns) by EUROPattern in up to 74.2% of the samples (collective B). This rate appears moderate, but is fairly high considering the complex system requirements. To further improve the otherwise very good performance characteristics (e.g., sensitivity) of EUROPattern and other automated systems [18], the current deficiencies in differentiating mixed patterns and in identifying some particular antibody reactivities [17, 18, 21] have to be overcome. For this purpose, further software development will enable the classification of a larger variety of diagnostically relevant cell and tissue fluorescence patterns. Titering of the samples (at least two dilutions) is recommended to facilitate the interpretation of mixed patterns. Moreover, the concept of EUROPattern includes a short final step of approving positive results, in which the investigator can confirm, modify, or further detail the reported antibody patterns (if necessary). Based on this concept, the performance of the automated approach potentially increases to 100%, resulting in a system that provides highly efficient, fast, and standardized IIF ANA processing and evaluation. Accordingly, EUROPattern can be regarded as a powerful alternative to the conventional visual approach.

The results of the present study were obtained by manual assay incubation. Since the EUROPattern classificator is plugged into the Laboratory Management System EUROLabOffice, the available automated incubation systems can be integrated seamlessly into the IIF workflow process using the EUROPattern Suite. 

For all of the 351 samples in this study, the EUROPattern Microscope delivered extremely sharp, high-resolution images which are a prerequisite for image processing and computer-aided diagnosis. Counterstaining not only provides solid nucleus finding and mitosis identification, but also ensures that a potentially failed focus will never lead to a false negative result.

Large laboratories with a high sample throughput tend to have a two-step IIF diagnostic process. Positive/negative screening is performed with a particular screening dilution (e.g., 1 : 80 or 1 : 100). Further dilutions are carried out for positive samples. EUROPattern merges the results from all available dilutions into one final result per patient, which is displayed together with all IIF images on a single patient-specific report form. The batchwise verification of negatives significantly shortens the analysis procedure.

Considering economic constraints and the growing demand for ANA detection in clinical practice, the system's unique capacity of slide accommodation (500 reaction fields) and high throughput (approximately 60 min for 100 reaction fields, depending on customer-specific settings) is of practical relevance, enabling the rapid processing of large sample quantities and overnight runs. Due to the casing around the magazine and microscope stage, the substrates are protected from bleaching and microscopy can be performed under normal room light conditions without need for a darkroom. The complete microscopic process, the acquisition of focused images, the management, processing, and archiving of data and images, is carried out by the system. EUROPattern recognizes most of the important ANA patterns, including mixed patterns, and calculates all corresponding titers. A diagnostic expert then performs the final validation of results at the office PC and may additionally access the system via a microscope control device. The EUROPattern Suite is in a continuous development process, which focusses on an even greater variety of fluorescence patterns and on several other features that will improve work processes, performance, and accuracy.

Certainly, the serodiagnosis of other autoantibody-associated diseases would also benefit from the implementation of automated IIF evaluation. For this purpose, the system will be applied to further antigenic substrates, such as neutrophil granulocytes in the detection of antineutrophil cytoplasmic antibodies in Wegener's granulomatosis, microscopic polyangiitis, and Churg-Strauss syndrome [24]. 

## 5. Conclusions

Compared to conventional visual IIF evaluation, EUROPattern proved to be very sensitive. This reliability is the basis for handing over the first step in ANA screening to an automated detection system. EUROPattern also proved to be highly efficient in sorting out negatives and providing good pattern recognition. The remaining process of validating positive results, which is carried out by qualified laboratory personnel patient by patient in the EUROPattern GUI, is now less time consuming and less error prone than direct visual reading. It can be expected that the intra- and inter-laboratory variation in IIF evaluation will be reduced efficiently by automation solutions, helping clinical laboratories to standardize IIF-based ANA diagnostics.

## Figures and Tables

**Table 1:** Comparison of software-based and visual positive/negative classification.

	Visual evaluation					
	Collective A (*n* = 200)		Collective B (*n* = 151)		Total (*n* = 351)	
	Positive	Negative	Positive	Negative	Positive	Negative
EUROPattern						
Positive	193	1	79	1	272	2
Negative	0	6	0	71	0	77
Concordance	99.5%		99.3%		99.4%	
*κ*-value	0.921		0.987		0.984	
Sensitivity	100%		100%		100%	
Specificity	85.7%		98.6%		97.5%	
PPV	99.5%		98.8%		99.3%	
NPV	100%		100%		100%	

*κ*: kappa-value indicating interrater agreement, PPV: positive predictive value, NPV: negative predictive value.

**Table 2:** Main pattern recognition by EUROPattern.

	EUROPattern performance					
Main ANA pattern	Collective A (*n* = 200)		Collective B (*n* = 151)		Total (*n* = 351)	
	No. of samples	Pattern recognized	No. of samples	Pattern recognized	No. of samples	Pattern recognized
Homogenous	24	20 (83.3%)	9	7 (77.8%)	33	27 (81.8%)
Speckled	94	90 (95.7%)	36	33 (91.7%)	130	123 (94.6%)
Nucleolar	18	17 (94.4%)	27	26 (96.3%)	45	43 (95.6%)
Centromeres	3	3 (100%)	1	1 (100%)	4	4 (100%)
Nuclear dots	1	1 (100%)	1	1 (100%)	2	2 (100%)
Cytoplasmic	53	49 (92.5%)	5	5 (100%)	58	54 (93.1%)
Negative	7	6 (85.7%)	72	71 (98.6%)	79	77 (97.5%)

Total	200	186 (93.0%)	151	144 (95.4%)	351	330 (94.0%)
